# Supplementary material for: Hypothalamic hormone deficiency enables physiological anorexia in ground squirrels during hibernation
Source: Nat Commun. 2024 Jul 10;15:5803. doi: 10.1038/s41467-024-49996-2 (PMC11236985; doi:10.1038/s41467-024-49996-2)
Supplement: Supplementary file 1 — Supplementary Information [file 41467_2024_49996_MOESM1_ESM.pdf]

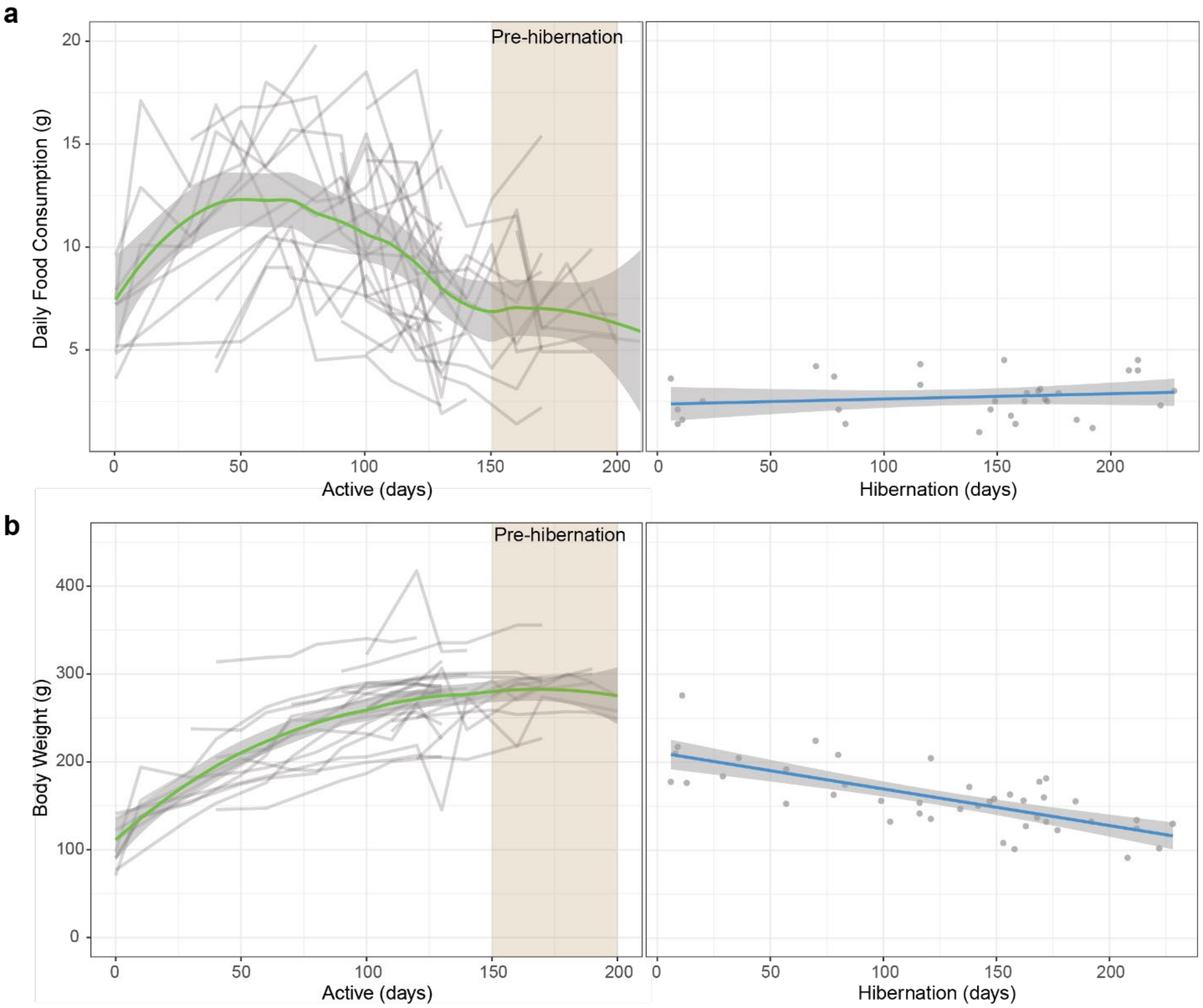

Extended Data Fig. 1. Seasonal feeding and body weight changes.
Daily food consumption (**a**) and body weight (**b**) of squirrels plotted by days active and days in hibernation. Food consumption and body weight were assessed longitudinally within animals across the active season into pre-hibernation, with each line representing one squirrel (*n* = 25 active and pre-hibernation animals). IBA feeding and body weight were assessed only once per hibernation season per squirrel, with each point representing one squirrel (*n* = 36 IBA animals for food measurement and *n* = 42 animals for body weight measurement). Active data was fit with a loess regression and hibernating data was fit with a simple linear regression. Source data are provided as a Source Data file.

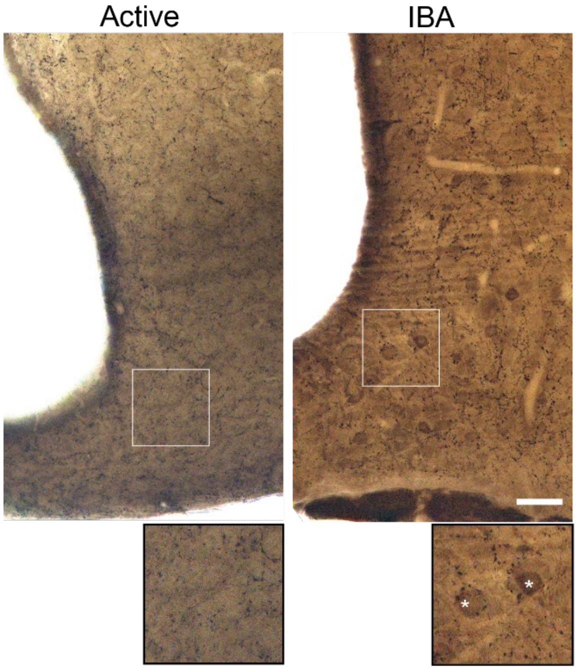

Extended Data Fig. 2. Immunoelectron microscopy of AgRP neurons.
Representative immunoelectron microscopy images of the arcuate nucleus across states demonstrating AgRP+ staining in the neuronal somas of IBA neurons only. Asterisks indicate neuronal soma. Scale bar = 50 μm.

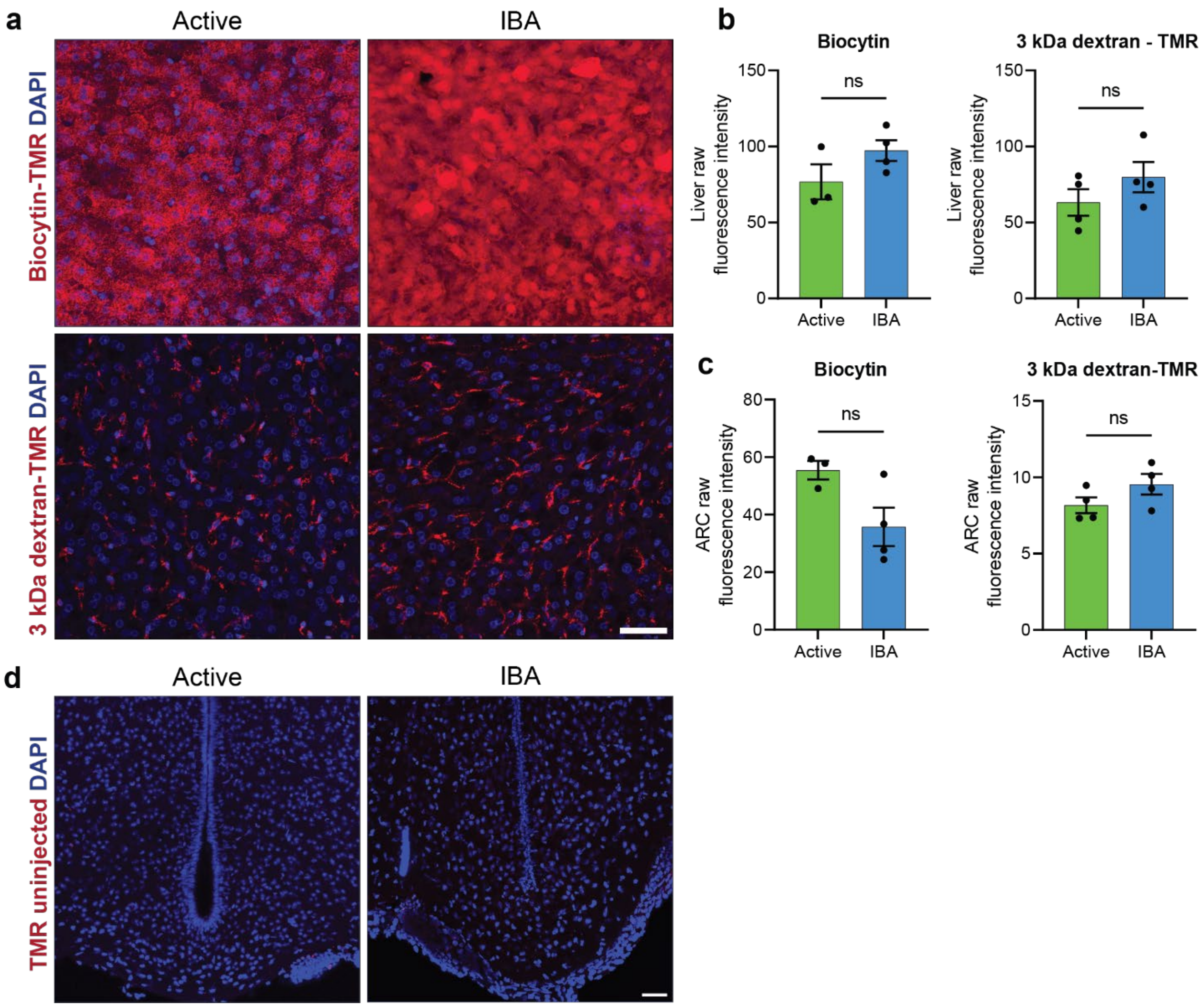

Extended Data Fig. 3. Raw fluorescence and uninjected controls for blood brain barrier permeability assays.

a, Representative immunohistochemistry images of liver across states after tail artery injection of 860 Da biocytin-TMR and 3kDa dextran-tetramethylrhodamine (TMR).

b - c, Raw fluorescence intensity of dye in (**b**), liver and arcuate nucleus and (**c**), median eminence across states (mean ± SEM, each point represents one animal, dextran: n = 4 and n = 4 IBA animals; biocytin: n = 3 active and n = 4 IBA animals, student's t-test, P > 0.05). Note that representative immunohistochemistry images of the ARC-ME after dye injection are shown in Main Fig 4h.

d, Representative images demonstrating lack of fluorescence in animals that were not injected with dye.

Scale bars, 50 µm. Source data are provided as a Source Data file.

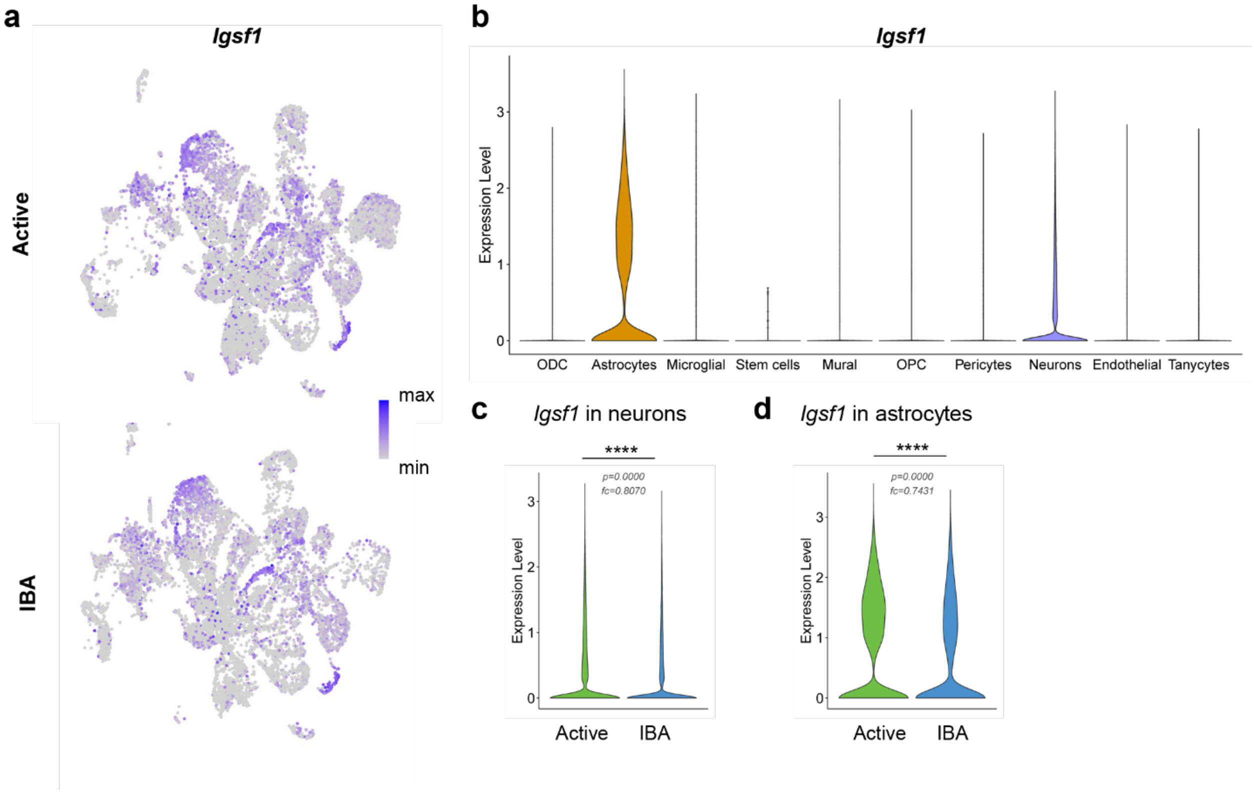

Extended Data Fig. 4. IGSF1 is downregulated in ARC neurons during hibernation

a, 2-dimensional UMAP projection of *Igsf1* gene expression in individual arcuate nucleus and median eminence (ARC-ME) cells (normalized, log-transformed, and represented by color as indicated in the color bar).

b, Violin plots showing expression of *Igsf1* in identified cell types aggregated for both states.

c, d, Expression of *Igsf1* across states in (**c**), all neurons and (**d**), astrocytes. Violin plots show normalized log-transformed gene counts. (Wilcoxon rank sum test (R/Seurat)). ****$P < 0.0001$; fc = fold change.

Cluster	NumCells	PercentCells	Marker1	Marker2	Marker3
0	3766	11.93	NRXN3	LRRTM4	DCC
1	2916	9.24	TENM2	LDB2	CLSTN2
2	2246	7.11	TAC1	ADCYAP1	PENK
3	1912	6.06	POMC	CARTPT	SHISAL2B
4	1744	5.52	NPY	AGRP	CARTPT
5	1678	5.32	GRIK1	SGCZ	NEK10
6	1450	4.59	KISS1	TAC3	ESR1
7	1429	4.53	GAD2	TCF7L2	ALCAM
8	1419	4.49	TRHDE	RELN	GAL
9	1377	4.36	C8orf34	SATB2	TH
10	1334	4.23	TRPM3	PDE7B	LAMA3
11	1237	3.92	ENSSTOG00000024849	SLC1A3	CST3[mh]
12	998	3.16	TRH	SST	NTS
13	992	3.14	SLC4A4	SLC6A11	SPARCL1
14	895	2.84	PDE3A	SPHKAP	PPP1R17
15	731	2.32	GHRH	GAL	NKAIN3
16	710	2.25	APOD	PTGDS	DCN
17	662	2.1	NTS	CACNA2D3	NXPH1
18	560	1.77	PMCH	HCRT	GAL
19	548	1.74	PPP1R17	CCK	PDYN
20	547	1.73	CGA	CHGA	KRT27
21	521	1.65	CSRP2	OTP	HTATSF1
22	455	1.44	PDGFRA	FABP7	TF
23	367	1.16	MKX	NCALD	TMSB4X
24	342	1.08	TAC3	GAL	IGSF1
25	292	0.92	IGFBP5	RBPMS	MGP
26	254	0.8	TAC1	FOXP2	EPHA6
27	123	0.39	AVP	OXT.1[mh]	OXT[mh]
28	64	0.2	C1QA	C1QC	TYROBP

Extended Data Table 1. Top markers of neuronal clusters.
Top three markers of the 29 identified neuronal clusters from single-cell sequencing of ARC-ME neurons aggregated from active and IBA animals.
