# Supplementary material for: Hypothalamic hormone deficiency enables physiological anorexia in ground squirrels during hibernation
Source: Nat Commun. 2024 Jul 10;15:5803. doi: 10.1038/s41467-024-49996-2 (PMC11236985; doi:10.1038/s41467-024-49996-2)
Supplement: Supplementary file 3 — Reporting Summary [file 41467_2024_49996_MOESM3_ESM.pdf]

nature portfolio

Corresponding author(s): Sviatoslav Bagriantsev and Elena Gracheva

Last updated by author(s): Jun 7, 2024

Reporting Summary

Nature Portfolio wishes to improve the reproducibility of the work that we publish. This form provides structure for consistency and transparency in reporting. For further information on Nature Portfolio policies, see our Editorial Policies and the Editorial Policy Checklist.

Statistics

For all statistical analyses, confirm that the following items are present in the figure legend, table legend, main text, or Methods section.

n/a	Confirmed	
☐	☒	The exact sample size (*n*) for each experimental group/condition, given as a discrete number and unit of measurement
☐	☒	A statement on whether measurements were taken from distinct samples or whether the same sample was measured repeatedly
☐	☒	The statistical test(s) used AND whether they are one- or two-sided<br>*Only common tests should be described solely by name; describe more complex techniques in the Methods section.*
☐	☒	A description of all covariates tested
☐	☒	A description of any assumptions or corrections, such as tests of normality and adjustment for multiple comparisons
☐	☒	A full description of the statistical parameters including central tendency (e.g. means) or other basic estimates (e.g. regression coefficient) AND variation (e.g. standard deviation) or associated estimates of uncertainty (e.g. confidence intervals)
☐	☒	For null hypothesis testing, the test statistic (e.g. *F*, *t*, *r*) with confidence intervals, effect sizes, degrees of freedom and *P* value noted<br>*Give P values as exact values whenever suitable.*
☒	☐	For Bayesian analysis, information on the choice of priors and Markov chain Monte Carlo settings
☒	☐	For hierarchical and complex designs, identification of the appropriate level for tests and full reporting of outcomes
☒	☐	Estimates of effect sizes (e.g. Cohen's *d*, Pearson's *r*), indicating how they were calculated

Our web collection on statistics for biologists contains articles on many of the points above.

Software and code

Policy information about availability of computer code

Data collection

LASX software was used to collect confocal images. SoftMaxPro software was used to read absorbance values for ELISAs.

Data analysis

Statistical analyses were performed in GraphPad Prism v9.0 or higher (GraphPad Software, San Diego, CA) for all comparisons with the exception of single cell sequencing analysis, which was performed in R 4.2.1. Final figures were assembled in Adobe Illustrator.

For single-cell RNA sequencing analysis:
Raw sequencing reads were processed using 10X CellRanger v.6.1.2 (10X Genomics, Pleasanton, CA). Genome reference for thirteen-lined ground squirrel (Ictidomys tridecemlineatus) was built based on the reference genome sequence and annotation obtained from the Ensembl project (www.ensembl.org2 Release 101; all files accessed on 11/20/2020):
Genome:
ftp://ftp.ensembl.org/pub/release-101/fasta/ictidomys_tridecemlineatus/dna/Ictidomys_tridecemlineatus.SpeTri2.0.dna.toplevel.fa.gz
Annotation:
ftp://ftp.ensembl.org/pub/release-101/gtf/ictidomys_tridecemlineatus/Ictidomys_tridecemlineatus.SpeTri2.0.101.gtf.gz
Read count matrix was further processed using R 4.2.1, RStudio 2022.02.3, and Seurat 4.1.13.

Image analysis was performed in ImageJ.

For manuscripts utilizing custom algorithms or software that are central to the research but not yet described in published literature, software must be made available to editors and reviewers. We strongly encourage code deposition in a community repository (e.g. GitHub). See the Nature Portfolio guidelines for submitting code & software for further information.

Data

Policy information about availability of data

All manuscripts must include a data availability statement. This statement should provide the following information, where applicable:

- Accession codes, unique identifiers, or web links for publicly available datasets
- A description of any restrictions on data availability
- For clinical datasets or third party data, please ensure that the statement adheres to our policy

All data are available in the main text or the supplementary materials. The RNA sequencing data was deposited to the Gene Expression Omnibus, accession number: GSE242381. Source data are provided with this paper.

Research involving human participants, their data, or biological material

Policy information about studies with human participants or human data. See also policy information about sex, gender (identity/presentation), and sexual orientation and race, ethnicity and racism.

Reporting on sex and gender	N/A
Reporting on race, ethnicity, or other socially relevant groupings	N/A
Population characteristics	N/A
Recruitment	N/A
Ethics oversight	N/A

Note that full information on the approval of the study protocol must also be provided in the manuscript.

Field-specific reporting

Please select the one below that is the best fit for your research. If you are not sure, read the appropriate sections before making your selection.

☒ Life sciences ☐ Behavioural & social sciences ☐ Ecological, evolutionary & environmental sciences

For a reference copy of the document with all sections, see nature.com/documents/nr-reporting-summary-flat.pdf

Life sciences study design

All studies must disclose on these points even when the disclosure is negative.

Sample size	No statistical method was used to predetermine sample size. Sample size was determined in accordance to standard practices in this field of research and was maximized based on the constraints of animal availability and seasonality. Immunohistochemistry data are quantified by animal and by section for completeness and contain at least n = 3 animals per group. Sample size for behavioral data contain at least n = 3 per paired groups and at least n = 4 per unpaired group. Sample size for blood metabolites and hormone analysis are at least n = 4 per group. Three biological replicates were utilized per group for sc-sequencing and the number of cells sequenced reported in the text.
Data exclusions	A ROUT outlier test (Q = 1 %) was run to identify one outlier in the Active state and two outliers in the IBA state for plasma leptin measurement (Fig 3a). Exclusion criteria were not pre-established, but given the spread of the data, we chose to use accepted methods to identify outliers. Before outlier exclusion, normality of the data was assessed by the Shapiro-Wilk normality test. The data failed the normality test and was thus analyzed using the nonparametric Mann-Whitney test. The p-value is disclosed in the figure for transparency and excluded values disclosed in source data.<br><br>The daily food consumption of hibernating animals (Fig 1c) whose body temperature dropped below 32°C within the 24 hour testing window were excluded from analysis, as defined by our criteria outlined in the methods for IBA animals. The body weight of excluded animals was included (Fig 1c).
Replication	Behavioral and blood hormone/ metabolite studies were replicated across at least two hibernation and/or active seasons, with the number of biological replicates reported in the legends and/or text. Immunohistochemistry data were replicated with individual animals at least three times with sample size reported in the text. For sc-sequencing data collection, three biological replicates per group were included. All attempt at replication were successful.
Randomization	Individuals in experimental groups were chosen to best match body weight and to represent both sexes across all groups. Constraints on animal availability, season, and state further constrained the pool of candidates. Out of the pool of candidates that met the body weight ranges, sex, and state criteria, animals were allocated randomly.
Blinding	No blinding was used for behavioral data collection. Due to the unpredictable nature of IBAs, their short duration, and practical criteria (CBT,

Blinding	facility temperature, time of year etc) that had to be met, it was not possible to perform behavioral experiments blinded. Immunohistochemistry quantification was performed blinded.

Reporting for specific materials, systems and methods

We require information from authors about some types of materials, experimental systems and methods used in many studies. Here, indicate whether each material, system or method listed is relevant to your study. If you are not sure if a list item applies to your research, read the appropriate section before selecting a response.

Materials & experimental systems

n/a	Involved in the study
☐	☒ Antibodies
☒	☐ Eukaryotic cell lines
☒	☐ Palaeontology and archaeology
☐	☒ Animals and other organisms
☒	☐ Clinical data
☒	☐ Dual use research of concern
☒	☐ Plants

Methods

n/a	Involved in the study
☒	☐ ChIP-seq
☒	☐ Flow cytometry
☒	☐ MRI-based neuroimaging

Antibodies

Antibodies used	Primary antibodies for immunohistochemistry:<br>mouse monoclonal cFOS C-10, Santa Cruz, sc-271243, 1:500<br>rabbit polyclonal Phospho-Stat3 (Tyr705), Cell Signaling Technology, 9131, 1:200<br>porcine anti-rabbit polyclonal POMC, Phoenix Pharmaceuticals, H-029-030, 1:2000<br><br>Secondary antibodies for immunohistochemistry:<br>Alexa Fluor 488 goat anti-mouse, Invitrogen, a11001, 1:400<br>Alexa Fluor 555 goat anti-rabbit, Abcam, ab150086, 1:1000<br><br>Conjugated antibody for immunohistochemistry:<br>Streptavidin-AlexaFluor594 , ThermoFisher S11227, 1:1000<br><br>Primary antibody for immuno-EM:<br>rabbit anti-AgRP antibody, Phoenix Pharmaceuticals, 1:2000<br><br>Secondary antibody for immuno-EM:<br>biotinylated goat anti-rabbit IgG, Vector Laboratories Inc., 1:250
Validation	Commercial antibodies were validated by the supplier in species specified. For antibodies whose epitope was disclosed by the supplier, their amino acid sequences were compared against the sequence of the corresponding protein of thirteen-lined ground squirrel and showed a > 96% conservation (CFOS: 100%, POMC: 96%). Antibodies whose epitope sequence is propitiatory were chosen by use in the field and/or had cross-reactivity across multiple species (PMID: 32885528; PSTAT3 cross reactivity: Human, Mouse, Rat, Monkey)

Animals and other research organisms

Policy information about studies involving animals; ARRIVE guidelines recommended for reporting animal research, and Sex and Gender in Research

Laboratory animals	Thirteen-lined ground squirrels (Ictidomys tridecemlineatus) were obtained from Dr. Dana Merriman (University of Wisconsin-Oshkosh) and/or bred in our facilities (Yale University). Animals (age 0.5 – 3 years) of both sexes were single housed in temperature- and humidity-controlled facilities at Yale University.
Wild animals	All animals utilized in this study were born and/or bred in captivity.
Reporting on sex	Similar proportions of male and females were used in each experiment, with the exception of single-cell RNA sequencing. Single-cell RNA sequencing was performed on male animals only in order to reduce unforeseen variability associated with sex on gene expression. For most experiments, sample size did not permit sex-specific analysis. For seasonal feeding and body weight (Fig 1c), sex specific analysis was performed and no statistical difference was seen for food consumed or body weight. Sex is reported for these data in the Source Data file.
Field-collected samples	This study did not include field-collected samples.
Ethics oversight	All experimental procedures were performed in compliance with the Institutional Animal Care and Use Committee of Yale University (protocol 2021-11497).

Note that full information on the approval of the study protocol must also be provided in the manuscript.

Plants

Seed stocks	N/A
Novel plant genotypes	N/A
Authentication	N/A
